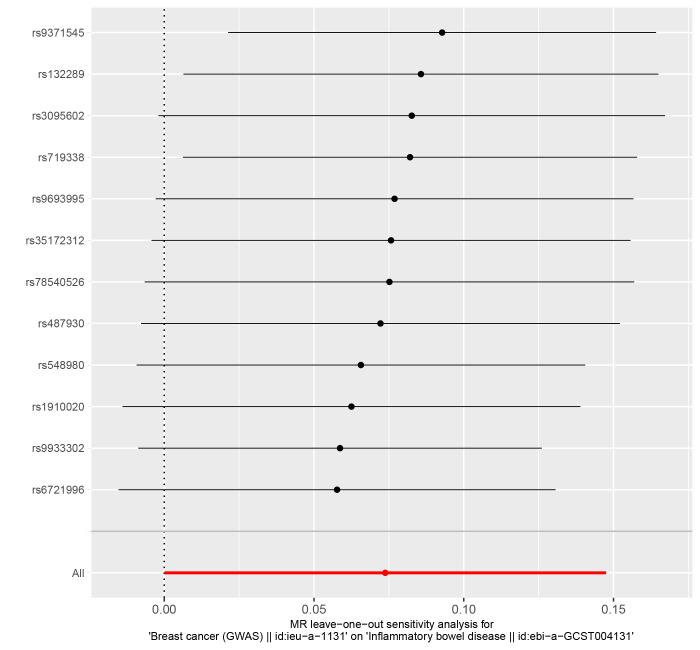# Inflammatory bowel disease and breast cancer: A two-sample bidirectional Mendelian randomization study

**DOI:** 10.1097/MD.0000000000038392

**Published:** 2024-06-07

**Authors:** Zihao Guo, Changyu Xu, Zhihao Fang, Xiaoxiao Yu, Kai Yang, Changxu Liu, Xinwei Ning, Zhichao Dong, Chang Liu

**Affiliations:** a Department of General Surgery, Fourth Affiliated Hospital of Harbin Medical University, Harbin, China; b Department of Ultrasound, Fourth Affiliated Hospital of Harbin Medical University, Harbin, China.

**Keywords:** breast cancer, Crohn disease, inflammatory bowel disease, Mendelian randomization, ulcerative colitis

## Abstract

There is a correlation between IBD and breast cancer according to previous observational studies. However, so far there is no evidence to support if there is a causal relationship between these 2 diseases. We acquired comprehensive Genome-Wide Association Study (GWAS) summary data on IBD (including ulcerative colitis [UC] and Crohn disease [CD]) as well as breast cancer of completely European descent from the IEU GWAS database. The estimation of bidirectional causality between IBD (including UC and CD) and breast cancer was achieved through the utilization of 2-sample Mendelian randomization (MR). The MR results were also assessed for any potential bias caused by heterogeneity and pleiotropy through sensitivity analyses. Our study found a bidirectional causal effect between IBD and breast cancer. Genetic susceptibility to IBD was associated with an increased risk of breast cancer (OR = 1.053, 95% CI: 1.016–1.090, *P* = .004). Similarly, the presence of breast cancer may increase the risk of IBD (OR = 1.111, 95% CI: 1.035–1.194, *P* = .004). Moreover, the bidirectional causal effect between IBD and breast cancer can be confirmed by another GWAS of IBD. Subtype analysis showed that CD was associated with breast cancer (OR = 1.050, 95% CI: 1.020–1.080, *P* < .001), but not UC and breast cancer. There was a suggestive association between breast cancer and UC (OR = 1.106, 95% CI: 1.011–1.209, *P* = .028), but not with CD. This study supports a bidirectional causal effect between IBD and breast cancer. There appear to be considerable differences in the specific associations of UC and CD with AD. Understanding that IBD including its specific subtypes and breast cancer constitute common risk factors can contribute to the clinical management of both diseases.

## 1. Introduction

Inflammatory bowel disease (IBD) is a long-term gastrointestinal condition characterized by inflammation, which includes Crohn disease (CD) and ulcerative colitis (UC), whose symptoms evolve with recurrence and remission. Clinical manifestations are dominated by gastrointestinal symptoms including diarrhea, gastrointestinal bleeding, and abdominal pain, but may also include extraintestinal manifestations of the skin, mucous membranes, joints, eyes, liver, and gallbladder.^[1]^ Patients with IBD may suffer from a lifelong threat of inflammation and related consequences, one of which is an increased risk of developing cancer.^[2,3]^ The most common type of cancer in IBD is colorectal cancer.^[4]^ However, the risk of cancer in other organs, such as lymphoma, lung, cervical and liver cancer, is also increased.^[5]^

Breast cancer has surged to the forefront of the worldwide cancer incidence and cancer mortality rates among women in 2020, accounting for 15.5% of cancer-related deaths in women.^[6]^ Some evidence suggests that chronic, systemic, or local inflammation is a driving factor in breast cancer formation, recurrence, and mortality.^[7,8]^ Nevertheless, the absence of dependable evidence regarding the potential impact of chronic inflammation caused by IBD on breast cancer persists. One study evaluating the prevalence of all malignancies in first-degree relatives of patients with CD found that the occurrence of breast cancer in first-degree relatives of patients with CD was more than twice as high as that of controls.^[9]^ In contrast, the authors found a significant reduction in breast cancer risk in the overall CD population, whereas the overall UC population had a higher risk of breast cancer relative to the normal population, but the difference was not significant.^[10]^ A recent meta-analysis showed no significant effect of IBD on breast cancer.^[11]^ Therefore, the relationship between breast cancer and IBD has been disputed by studies with similar purposes. No proof exists to suggest a causal relationship between the 2 diseases. Further conclusive studies on the risk of breast cancer in patients with IBD are needed.

**Figure 1.**
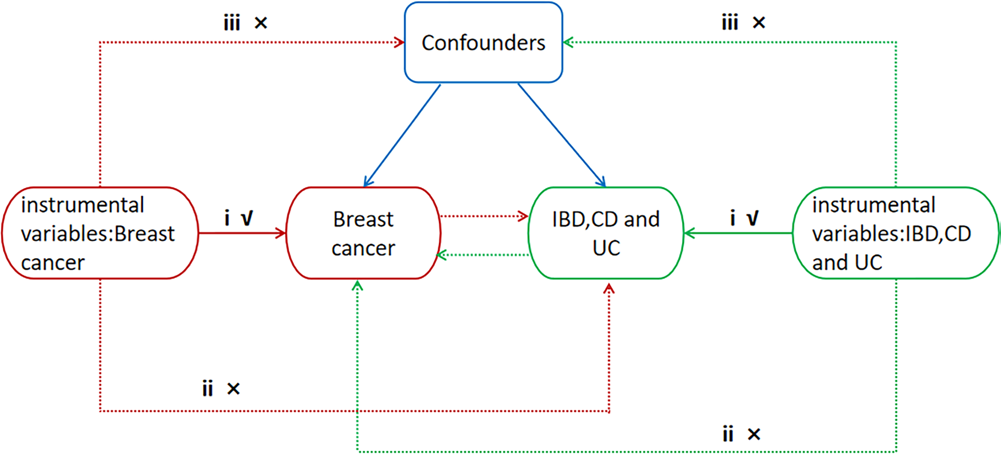
Design of the Mendelian randomization study. (i) The instrumental variables were significantly associated with exposure. (ii) The instrumental variables were not associated with any potential confounders. (iii) The instrumental variables can only affect outcomes through exposure. CD = Crohn disease, IBD = inflammatory bowel disease, UC = ulcerative colitis.

Mendelian randomization (MR) is a technique that utilizes genetic variants as instrumental variables (IVs) to determine the causal link between exposure and outcome.^[12]^ With the continuous expansion of MR research methodology, it has been used as a more desirable research method at the gene level. The MR analysis uses SNPS as IVs and the random, independent assignment of alleles during meiosis. In addition, the genetic information is fixed at the time of zygote formation, which is not affected by later environmental factors and the direction of causality is determined.^[13]^ Therefore, it can avoid some limitations of causal inference in observational studies (confounding, reverse, causation, and regression dilution bias) and randomized controlled studies (expensive, time-consuming, and ethical), and has been gradually applied to the inference of pathogenetic associations between 2 complex diseases.^[14]^ No MR studies have investigated the causal relationship between IBD and breast cancer. Therefore, we performed MR analysis of 2 samples with the aim of exploring the potential bidirectional relationship between IBD, its subtypes and breast cancer, and providing useful recommendations for clinical practice. The flow chart of this study is shown in Figure 1.

## 2. Methods

### 2.1. Data sources and study design

MR is a method to analyze the causal relationship between exposure and outcome by using genetic variants as IVs. Three core assumptions should be satisfied: Correlation hypothesis: there is a strong correlation between genetic variation and exposure factors. Independence hypothesis: genetic variation is not affected by confounding factors such as environment. Pleiotropy hypothesis: Genetic variation can only affect the outcome through exposure factors, but not through other ways. In other words, there is no horizontal pleiotropy.

In order to mitigate the impact of population stratification, all SNPs and their corresponding summary data were acquired from studies exclusively encompassing populations of European descent. We downloaded all IVs involved in the MR analysis from the IEU Genome-Wide Association Study (GWAS) database (https://gwas.mrcieu.ac.uk/datasets/). SNPs associated with IBD were obtained from the International Inflammatory Bowel Disease Genetics Consortium, selected from the recent GWAS meta-analysis of IBD by Liu et al.^[15]^ The analysis included IBD (12,882 cases; 21,770 controls), CD (5956 cases; 14,927 controls) and UC (6968 cases; 20,464 controls). The diagnosis of IBD is based on recognized radiological, endoscopic, and histopathological evaluations. Another large GWAS summary of IBD data by de Lange et al was used as a validation analysis, with 25,042 IBD cases and 34,915 controls.^[16]^ The study obtained GWAS summary statistics from the Breast Cancer Association Consortium (BCAC) for a total of 14,910 cases of breast cancer and 17,588 controls.^[17]^

### 2.2. Instrumental variables selection and quality control

Firstly, we extracted SNPs of genome-wide significance associated with exposure (*P* < 5 × 10-8). Secondly, to be unaffected by strong linkage disequilibrium (LD), we set the threshold for LD to *R*^2^ < 0.001 and the physical distance between genes kb = 10,000. Thirdly, we eliminated SNPs that had a minor allele frequency lower than 0. 01. Fourthly, when the desired SNPs were not present in the outcomes of the research, we employed proxy SNPS (high LD with *R*^2^ > 0.8 of the target SNPS) as replacements. Finally, in order to harmonize exposure and outcome SNPs, palindromes and incompatible alleles were removed.

In addition, weak IVs may affect the results, so we used the F score to assess whether the selected SNPs were affected by weak IVs. The formula was F = (F = β^2^ exposure/SE^2^exposure). If the F statistic of SNP is <10, it will be excluded from the further analysis.^[18]^

### 2.3. Statistical analyses

The main analysis employed the inverse variance weighted (IVW) method. In the absence of heterogeneity, the fixed effects method is employed; conversely, the random effects method is utilized.^[19]^ MR Egger and weighted median were also performed to estimate the causal effect of exposure on the outcome to make our results more robust. A meta-analysis was conducted to determine the causal relationship between exposure and outcome by utilizing the IVW method and comparing the SNP exposure effect to the SNP outcome effect.^[20]^ The weighted median is more tolerant of genetic IVs, and only more than 50% of the IVs need to be valid IVs to obtain stable effect values.^[21]^ MR-Egger regression has the highest tolerance for horizontal pleiotropy. When none of the SNPS satisfy the MR hypothesis, it still provides a valid causal estimate.^[22]^

All MR analyses in this study were performed using the “TwoSample MR” package in the R software (version 4.3.1). A *P* value < .05 was considered to indicate statistical significance.

### 2.4. Heterogeneity and sensitivity test

Heterogeneity was assessed by the IVW method and quantified by Cochran Q statistic; *P* < .05 indicated the presence of heterogeneity.^[23]^ The detection of horizontal pleiotropy was achieved through the utilization of MR-Egger regression. The MR-Egger method intercept value indicates the intensity of horizontal pleiotropy; a *P* value > .05 implies the absence of horizontal pleiotropy.^[24]^ MR-PRESSO detects SNPS that may cause a polyvalent effect. The estimates were reassessed after the outliers were removed.^[25]^ The leave-one-out method enhances the credibility of the results. This method gradually removes individual SNPs and performs MR analysis on the remaining SNPs to test whether individual SNPs significantly affect the results.^[26]^

## 3. Results

### 3.1. The causal effect of IBD on breast cancer

We included significant (*P* < 5E-08) and independent (*R*^2^ < 0.001, kb = 10,000) SNPs. Finally, 53,45, and 31 IVs for IBD, CD, and UC were carefully selected after removing palindromic sequences (Supplementary Table S1-S3, http://links.lww.com/MD/M719, http://links.lww.com/MD/M720, http://links.lww.com/MD/M721). For these IVs, all F-values were >10. These results suggest that the variables are eligible for the strong correlation assumption of MR and that instrumental bias is so minimal that it does not substantially influence the estimation of causal effects.

**Figure 2.**
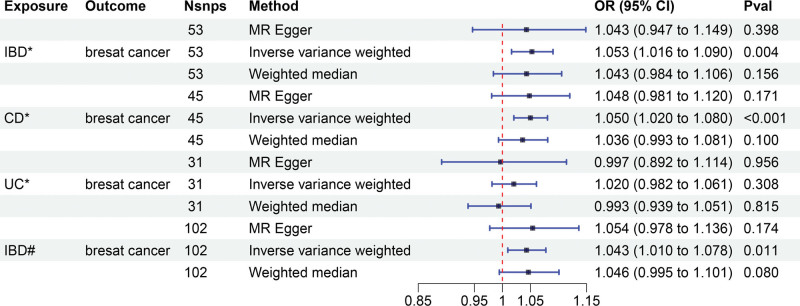
Mendelian randomization study for the causal effects of Inflammatory bowel disease on breast cancer. *Data from Liu et al. #Data from de Lange et al IBD = inflammatory bowel disease, CD = Crohn disease, UC = ulcerative colitis.

**Figure 3.**
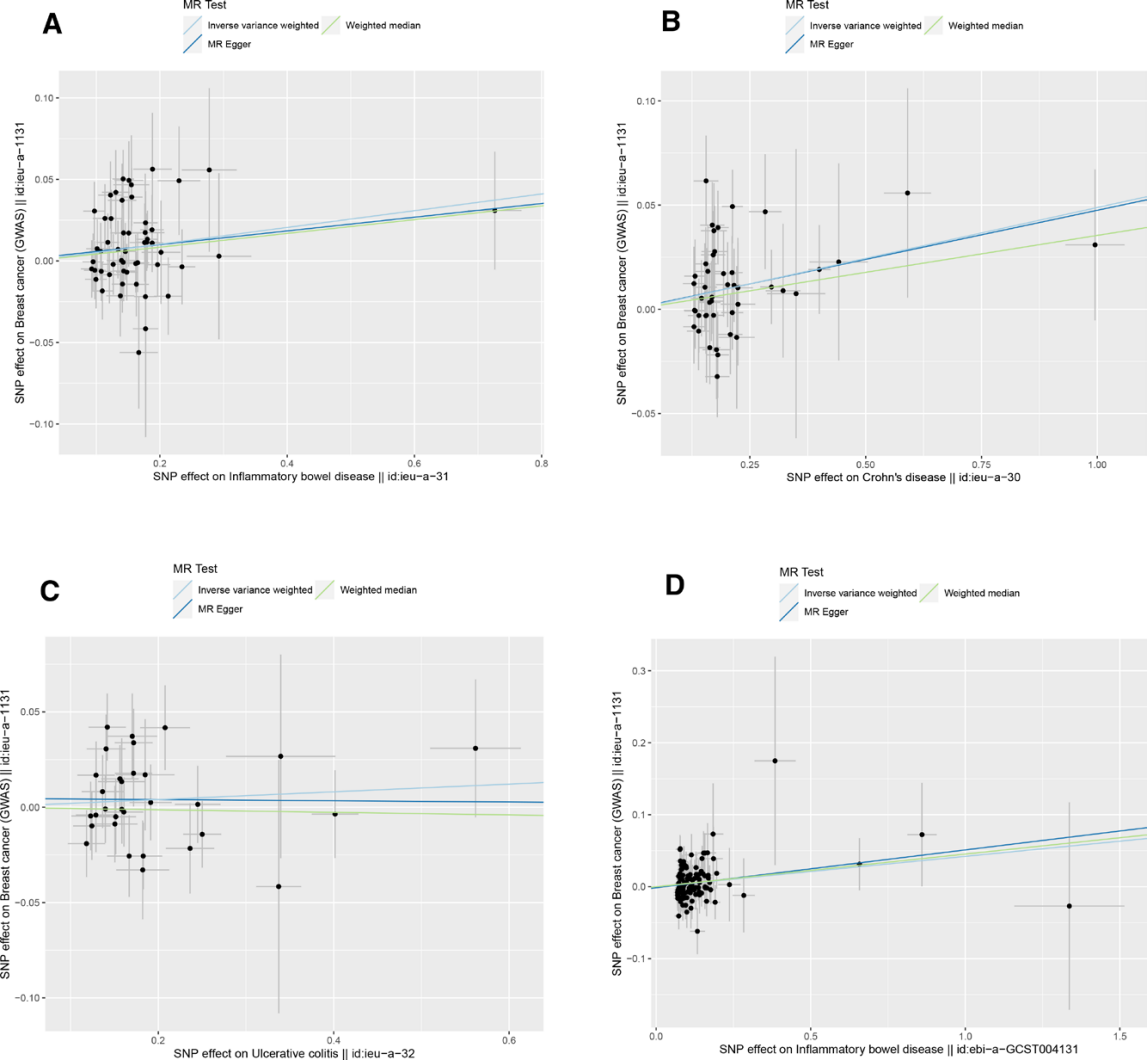
Scatterplot of causal effects of IBD (including CD and UC) on the risk of breast cancer by multiple Mendelian randomization methods. (A) IBD^*^ on breast cancer. (B) CD* on breast cancer. (C) UC* on breast cancer. (D) IBD# on breast cancer. *Data from Liu et al. #Data from de Lange et al IBD: inflammatory bowel disease, CD = Crohn disease, UC = ulcerative colitis.

The IVW (as the primary analysis method) approach showed a causal relationship between genetically predicted IBD and the risk of breast cancer (OR = 1.053, 95% CI: 1.016–1.090, *P* = .004). The MR-Egger regression and weighted median method showed no statistical differences (Fig. 2). Scatter plot (Fig. 3A) analysis demonstrates the results of MR analysis. The intercept term of MR-Egger (intercept = 0.002, *P* = .840), indicated that horizontal pleiotropy did not affect the results (Table 1). Cochran Q indicates no significant heterogeneity (*P* values of Cochran Q = 0.569) (Table 1). The leave-one-out sensitivity analysis indicated that the lack of a single SNP did not affect the causal correlation between IBD and the risk of breast cancer (Supplementary Figure S1, http://links.lww.com/MD/M711).

**Table 1:** Heterogeneity and pleiotropy analysis of genetically predicted inflammatory bowel disease on the risk of breast cancer.

Exposure	Outcome	Heterogeneity		Pleiotropy		MRPRESSO global test derived *P* value
		Heterogeneity (*P* value)	Heterogeneity (Q)	*P* value	Egger intercept	
IBD*	Breast cancer	.569	49.598	.840	0.002	.678
CD*	Breast cancer	.681	39.098	.956	0.001	.708
UC*	Breast cancer	.420	30.905	.663	0.005	.481
IBD#	Breast cancer	.791	89.293	.765	−0.001	.696

* Data from Liu et al.

#Data from de Lange et al CD = Crohn disease, IBD = inflammatory bowel disease, UC = ulcerative colitis.

In addition, according to the IVW approach, we found that genetic susceptibility of CD was significantly positively associated with breast cancer (OR = 1.050, 95% CI: 1.020–1.080, *P* < .001) (Fig. 2). However, there was no significant association between UC and breast cancer (OR = 1.020, 95% CI:0.982–1.061, *P* = .308) (Fig. 2). Scatter plots are shown in Figure 3B and C. The stability of the results was confirmed by leave-one-out analysis (Supplementary Fig. S2-S3, http://links.lww.com/MD/M712, http://links.lww.com/MD/M713). When exploring the effect of CD (*P* values of Cochran Q = 0.681) and UC (*P* values of Cochran Q = 0.420) on the risk of breast cancer, we found no heterogeneity (Table 1). The MR-PRESSO global test had *P* values of 0.678 for IBD, 0.708 for CD, and 0.481 for UC when tested on breast cancer (Table 1).

We obtained 102 SNPS for IBD for validation analysis, all of which proved to be reliable IVs (Supplementary Table S4, http://links.lww.com/MD/M722). Genetically predicted IBD was significantly positively associated with breast cancer (OR = 1.043, 95% CI: 1.010–1.078, *P* = .011) (Fig. 2). The scatter plot (Fig. 3D) also showed that the slopes of the results between IBD and breast cancer analyzed by the different methods were positive, and the consistent correlation trend indicated that the results of our analyses were very reliable. In addition, the leave-one-out analysis showed that the overall estimate was not affected by any of the SNPs (Supplementary Fig. S4, http://links.lww.com/MD/M714). MR-Egger regression indicated that horizontal pleiotropy did not bias the results (intercept = −0.001, *P* = .765) (Table 1). Heterogeneity was not significant in validation analyses (Table 1).

### 3.2. The causal effect of breast cancer on IBD

**Figure 4.**
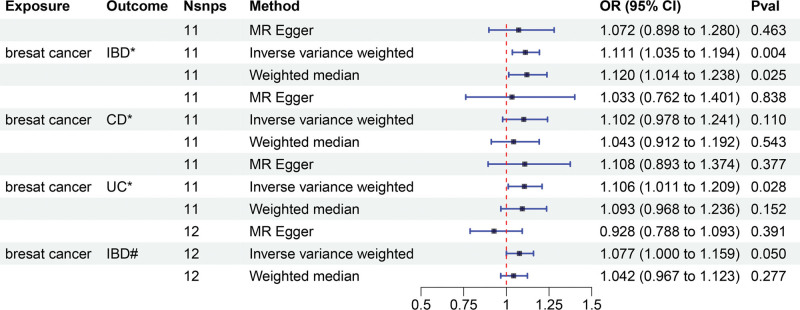
Mendelian randomization study for the causal effects of breast cancer on IBD. *Data from Liu et al. #Data from de Lange et al.

**Figure 5.**
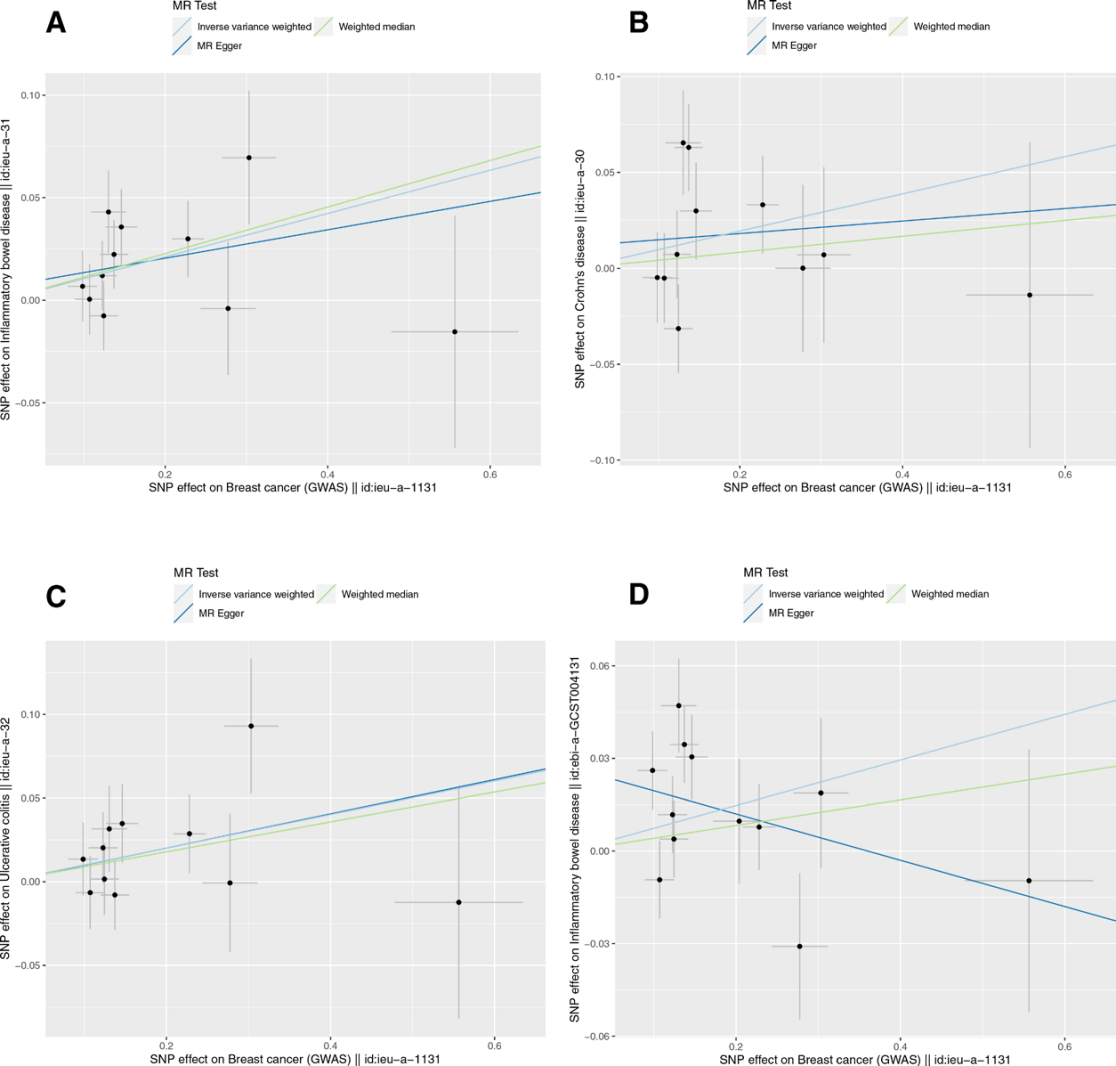
Scatterplot of causal effects of breast cancer on the risk of IBD (including CD and UC) by multiple Mendelian randomization methods. (A) Breast cancer on IBD*. (B) Breast cancer on CD*. (C) Breast cancer on UC*. (D) Breast cancer on IBD#. *Data from Liu et al. #Data from de Lange et al. IBD = inflammatory bowel disease, CD = Crohn disease, UC = ulcerative colitis.

In reverse MR Analysis, breast cancer was the exposure factor used to demonstrate IBD results. To explore the causal effects of breast cancer on IBD, CD, and UC, we included 11 significant and independent IVs. F statistics with IVs are all >10, indicating that there is no bias in the weak IVs (Supplementary Table S5-S7, http://links.lww.com/MD/M723, http://links.lww.com/MD/M724, http://links.lww.com/MD/M725). According to the IVW approach, there was a significant association between breast cancer and IBD (OR = 1.111, 95% CI: 1.035–1.194, *P* = .004) (Fig. 4). The causal effect of breast cancer on UC was statistically significant (OR = 1.106, 95% CI: 1.011–1.209, *P* = .028). We were unable to find any evidence of an association between breast cancer and CD (OR = 1.102, 95% CI = 0.978–1.241, *P* = .110). The forest plot is shown in Figure 4 and the scatter plot is shown in Figure 5. There is no significant heterogeneity in Cochran Q test (Table 2). We also used the GWAS data of IBD from de Lange et al to validate the reverse MR Analysis (Supplementary Table S8, http://links.lww.com/MD/M726). The IVW method heterogeneity test revealed a significant level of heterogeneity among genes (*P* values of Cochran Q = 0.018) (Table 2). The random effects method in IVW was chosen to eliminate the effects of heterogeneity as much as possible. The OR of IBD per genetically predicted 1-unit increase in log-transformed OR of breast cancer was 1.077 (OR = 1.077, 95% CI = 1.000–1.159, *P* = .050) (Fig. 4). The Egger test showed that there was no potential for horizontal pleiotropy in reverse MR analysis (Table 2). The stability of the results is proved by the leave-one method analysis (Supplementary Fig. S5-S8, http://links.lww.com/MD/M715, http://links.lww.com/MD/M716, http://links.lww.com/MD/M717, http://links.lww.com/MD/M718).

**Table 2:** Heterogeneity and pleiotropy analysis of genetically predicted breast cancer on the risk of Inflammatory bowel disease.

Exposure	Outcome	Heterogeneity		Pleiotropy		MRPRESSO global test derived *P* value
		Heterogeneity (*P* value)	Heterogeneity (Q)	*P* value	Egger intercept	
Breast cancer	IBD*	.471	9.658	.670	0.007	.485
Breast cancer	CD*	.136	14.905	.661	0.012	.157
Breast cancer	UC*	.695	7.315	.985	-0.001	.813
Breast cancer	IBD#	.018	22.940	.081	0.027	.028

* Data from Liu et al.

#Data from de Lange et al CD = Crohn disease, IBD = inflammatory bowel disease, UC = ulcerative colitis.

## 4. Discussion

The present study conducted a thorough analysis of the causal correlation between IBD and breast cancer, utilizing summarized GWAS data. As far as we know, this is the first study to use MR methods to assess bidirectional causality between IBD and breast cancer. We found a bidirectional positive causal relationship between IBD and breast cancer, which was confirmed using another GWAS data for IBD. In the subgroup analyses conducted on the 2 primary subtypes of IBD, it was found that genetically predicted CD exhibited an elevated risk of breast cancer, whereas UC did not display any such association. The results of the reverse MR analysis indicated that breast cancer had a considerable influence on UC, yet there was no evidence to suggest a causal link with CD.

Other preclinical and clinical studies provide evidence that IBD has a similar pathway to breast cancer in terms of occurrence and progression, rather than an unrelated link. A study examined the occurrence of breast cancer in family members of individuals with CD in contrast to family members of individuals without any signs of gastrointestinal illness. The findings indicate that CD has been recognized as an independent risk element for the emergence of breast cancer, and first-degree family members of individuals with CD are more prone to developing breast cancer. This could indicate an association between IBD and breast cancer, either hereditary or environmental.^[9]^ In our MR Analysis, genetically predicted CD was significantly positively associated with breast cancer (OR = 1.050, 95% CI: 1.020–1.080, *P* < .001). A large Norwegian study using data from the National Cancer Registry and the Centre for Cause-of-Death Studies aimed to determine the prevalence of intestinal parenteral cancer 20 years after diagnosis in an IBD cohort and to evaluate if these patients exhibited a heightened susceptibility to cancer in comparison to a corresponding control group. The results found that both UC and CD patients had a significantly increased risk of breast cancer compared to controls.^[27]^ Earlier research has indicated that the utilization of oral contraceptives and hormone replacement therapy pose a risk for IBD, both of which also impact breast cancer.^[28,29]^ In one of the included studies, the data showed that the risk of breast cancer was proportional to the frequency of hospitalization for IBD. In principle, patients in need of additional hospitalization would also be provided with more rigorous treatment to control disease activity.^[30]^ Thus, this finding suggests that IBD activity may be correlated with the risk of breast cancer. Our MR Analysis showed that genetically predicted CD and IBD increase the risk of breast cancer. This further supports the clinical studies mentioned above.

However, a recent meta-analysis including 16 cohort studies showed that IBD did not appear to have a significant effect on breast cancer risk, with an overall pooled OR of 0.94 (95% CI, 0.82–1.06) for patients with IBD. In further subgroup analyses, there was no significant correlation between breast cancer risk in patients with CD (OR, 0.91; 95% CI, 0.70–1.12) and UC (OR, 0.99; 95% CI, 0.90–1.08).^[11]^ One study was based on intestinal, parenteral, and overall cancer risk in an IBD cohort of the Dutch population. The authors found a reduced risk of breast cancer in the overall CD population (SIR 0.11; 95% CI 0.00–0.64), whereas the overall UC population had a high risk of breast cancer relative to the normal population, but the difference was not significant.^[10]^ The results of the epidemiologic studies remain controversial regarding the association between IBD and breast cancer. These controversial findings may be due to methodologic constraints and a limited number of patients. Moreover, observational studies may be subject to inevitable clinical confounders that can affect exposure and outcomes, impairing the ability of observations to make accurate causal judgments. Therefore, the results of previous observational studies on IBD and breast cancer may be biased. MR analysis can avoid the influence of these confounding factors by introducing genetic IVs, thus obtaining relatively accurate causal assessment.

The current MR study confirms a bidirectional causal relationship between IBD and breast cancer. In addition, our sub-analysis found an increased risk of breast cancer in patients with CD and that breast cancer was associated with UC. Although UC and CD subtypes occur in genetically susceptible individuals through interactions with environmental factors, there appear to be significant differences in the association of these subtypes with breast cancer. In an observational study examining the influence of IBD on the treatment and survival outcomes of breast cancer patients, it was discovered that breast cancer patients diagnosed with UC were administered identical treatment to those without IBD, and there were no discernible disparities in breast cancer survival rates between these individuals and those without IBD. However, breast cancer patients with CD receive less radiotherapy and more frequent chemotherapy than non-IBD patients, and breast cancer survival rates for CD patients receiving chemotherapy are worse than those for non-IBD patients.^[31]^ This further confirms our MR Results that CD but not UC confers an increased risk of breast cancer. Subtypes of the same large class of diseases may have some similarities in genetic background, but UC and CD still have significant differences in individual genes, gut microbiota, genetics, and immunity.^[32,33]^ Whether these differences lead to different associations of UC and CD with breast cancer needs to be clarified in future studies.

There are several mechanisms supporting the bidirectional relationship between IBD and breast cancer. Estrogen signaling is a key factor in controlling the development of breast cancer, which is mediated by estrogen receptors such as estrogen receptor alpha (ERα), estrogen receptor beta (ERβ), and G protein-coupled estrogen receptors (GPER). In line with breast cancer, elevated ERα levels, decreased ERβ levels, and increased GPER levels were also linked to the development of inflammatory activity in IBD.^[34,35]^ Another major molecule involved is breast cancer resistance protein (BCRP). It serves as a transporter for efflux, safeguarding intestinal cells against harmful substances and contributing to the transportation of bile acids in the intestines, as well as the progression of breast cancer. Gutmann et al discovered that patients with IBD exhibited a notable decrease in BCRP expression compared to control subjects without IBD.^[36]^ This suggests that IBD patients are at a higher risk of breast cancer. Japanese researchers speculate that there is a common genetic susceptibility to IBD and breast cancer and that it is associated with interleukin-1 (IL-1) polymorphisms.^[37]^ The alteration of MLH1 has been linked to vulnerability to IBD and HNPCC, a syndrome predisposing to parenteral malignancy.^[38,39]^ These data further support the hypothesis that there is a common genetic etiology between IBD and breast cancer.

Current studies suggest that there may be a common molecular mechanism between CD and breast cancer. Analysis of the gene Expression Omnibus repository public microarray expression data revealed 53 differentially expressed genes that overlapped between the CD group and the breast cancer group. Both diseases are associated with the IL-17 and NF-κB signaling pathways. Gene interaction network and module analysis showed that CXCL8, IL1β and PTGS2 were the hub genes.^[40]^ Inflammatory mediators such as CXCL8, IL1β, and PTGS2 may have a significant impact on cancer growth by controlling CSC proliferation and self-renewal.^[41]^ CXCL8 is also involved in neovascularization in the tumor microenvironment, which leads to tumor expansion and spread in the tumor microenvironment.^[42]^ IL-1β has been identified as a pro-inflammatory mediator that intensifies tumor-promoting inflammation in breast cancer.^[43]^ The β-catenin signaling pathway can be activated by IL-1β, leading to epithelial-mesenchymal transformation (EMT) in breast cancer cells.^[44]^ The induction of PGE2 by PTGS2 facilitates the migration and EMT of human breast cancer cells.^[45]^ Furthermore, the excessive expression of various inflammatory proteins (including CXCL8, IL1β, and PTGS2) in peripheral blood cells could potentially exacerbate systemic inflammation in women diagnosed with IBD.^[46]^

There is a notable disparity in the gut microbiota between individuals with IBD and those who are in good health, as confirmed by multiple studies.^[47]^ The composition and diversity of gut flora are key factors leading to the development of IBD.^[48,49]^ In the early stages of IBD, the composition of the gut microbiome may change. The intestinal flora composition fluctuates more in IBD patients than in healthy individuals. Compared with healthy controls, the level of *Bifidobacterium longum* in UC, beneficial bacteria such as *Eubacterium rectale, Faecalibacterium prausnitzii, Roseburia intestinalis* in CD and UC were significantly reduced. However, the relative abundance and growth rate of harmful bacteria such as *Bacteroides fragilis* increased.^[50]^ It is common for IBD patients to develop stronger antibody and T-cell responses to microbial antigens.^[51,52]^ Coordination of immune responses mediated by T cell differentiation subsets with intestinal flora may influence the development of IBD.^[53]^ A correlation was discovered between alterations in the gastrointestinal tract and the onset of breast cancer in mice, as indicated by a study. The alteration of the intestinal environment prompted an inflammatory reaction in the mice, leading to a modification in the growth pattern of breast cancer cells. The administration of antibiotics to ER-positive transplanted tumor mice disrupted the homeostasis of intestinal flora, leading to a systemic inflammatory response and the subsequent spread of the tumor.^[54]^ Furthermore, the bacteria in the intestines can make it difficult to respond to chemotherapy for breast cancer. Research conducted on mouse models and individuals diagnosed with HER2-positive breast cancer has demonstrated a direct correlation between intestinal flora and the effectiveness of trastuzumab. By introducing fecal bacteria from FMT-R mice into HER2 + breast cancer mice, the response to trastuzumab was replicated.^[55]^ These studies suggest that intestinal flora imbalance plays an important role in the pathogenesis of both IBD and breast cancer.

Studies have found that chronic psychological stress is associated with gastrointestinal dysfunction. The occurrence and subsequent recurrence of IBD are also linked to chronic psychosocial stress.^[56]^ Patients with persistent IBD or breast cancer alone have different levels of stress, while patients with IBD and breast cancer may experience greater stress. The inflammation in the intestines can affect the brain by activating the hypothalamic-pituitary-adrenal (HPA) axis. This can cause anxiety and depression in patients.^[57]^ The immune system anti-tumor immune response can be inhibited by these effects, potentially leading to an increased risk of breast cancer in individuals with CD.^[58]^

We want to highlight some of the advantages of our study and recognize certain restrictions. First, this is the first study to evaluate bidirectional causality between IBD and breast cancer using a 2-sample MR approach. Compared with observational studies, this method is less susceptible to confounding factors, reverse causality, and undifferentiated exposure. Second, we strictly define disease subtypes to avoid disease co-existence affecting outcomes. We found that CD and UC had fundamentally different relationships with breast cancer. Third, we conducted multiple sensitivity analyses to ensure consistency of causal estimates and robustness of results. Finally, we were able to validate the findings of the gene-predicted association between IBD and breast cancer by another largely independent GWAS of IBD, leading to similar causal estimates. However, we should also consider the limitations of this study. First, the inclusion of people of European ancestry in this study may limit the application of the findings to other ethnic populations. Second, the absence of publicly accessible source data hinders our ability to detect any potential bias in sample overlap. Finally, despite our efforts to remove any confounding variables, we cannot entirely dismiss the possibility of horizontal pleiotropy affecting our results.

## 5. Conclusion

In conclusion, our study suggests a bidirectional causality between IBD and breast cancer. Regarding the major subtypes of IBD, UC and CD, there appeared to be significant differences in their specific associations with breast cancer. Therefore, it is reasonable to recommend routine breast cancer screening in patients with IBD, and vice versa. Proper management of IBD may be important for reducing breast cancer risk, and vice versa. In addition, large-scale basic studies and clinical trials are needed to validate our findings and further elucidate the pathophysiology of the causal relationship between IBD and breast cancer.

## Author contributions

**Conceptualization:** Zihao Guo.

**Data curation:** Xinwei Ning.

**Formal analysis:** Xiaoxiao Yu.

**Funding acquisition:** Chang Liu.

**Investigation:** Changxu Liu.

**Methodology:** Changyu Xu.

**Resources:** Zhichao Dong.

**Software:** Zhihao Fang.

**Supervision:** Chang Liu.

**Validation:** Changyu Xu.

**Visualization:** Kai Yang.

**Writing – original draft:** Zihao Guo.

**Writing – review & editing:** Chang Liu.

## Supplementary Material







**Figure:**
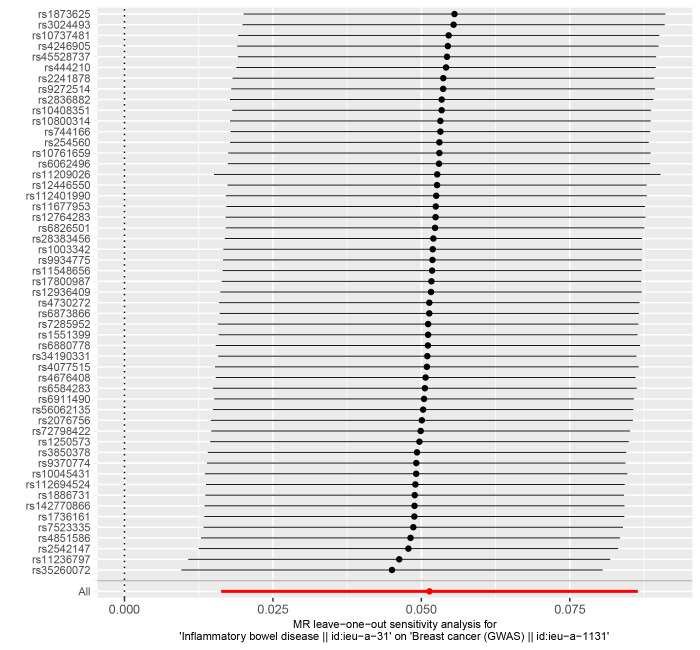


**Figure:**
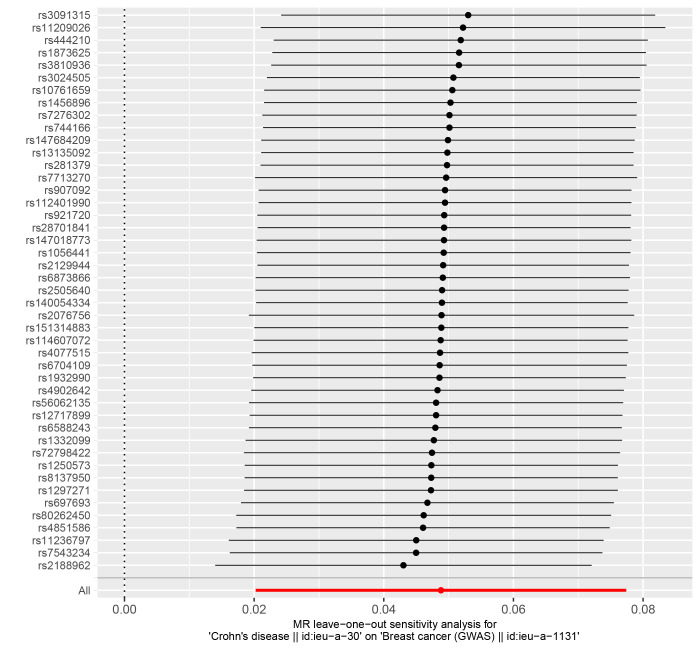


**Figure:**
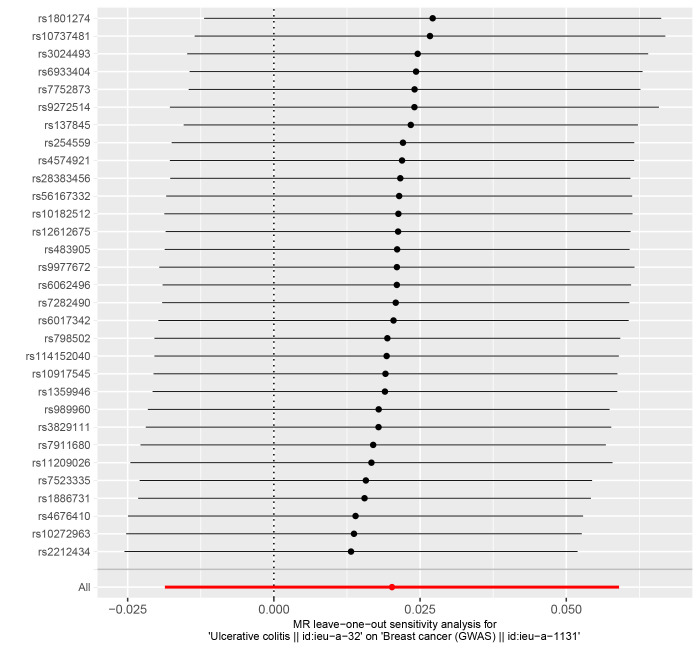




**Figure:**
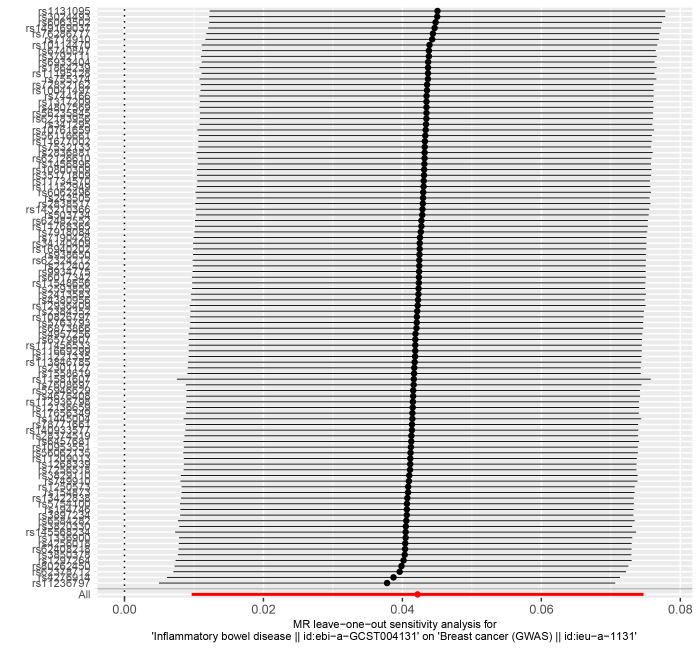










**Figure:**
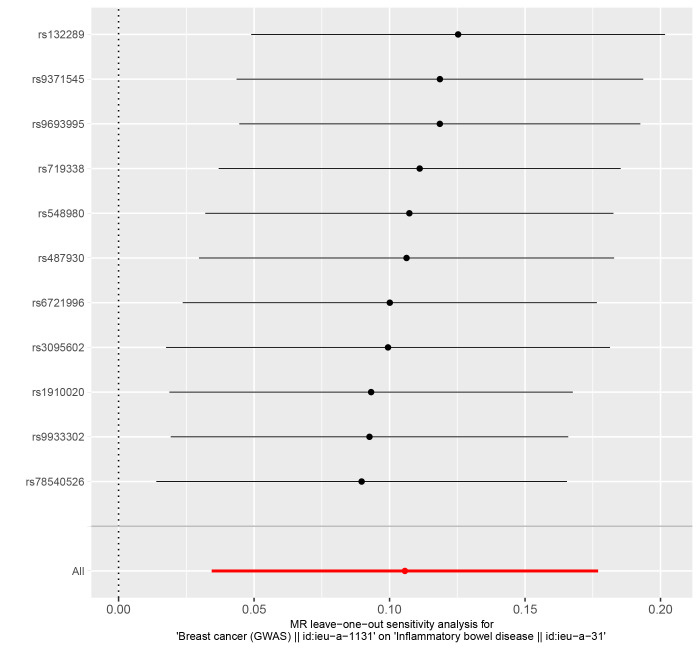


**Figure:**
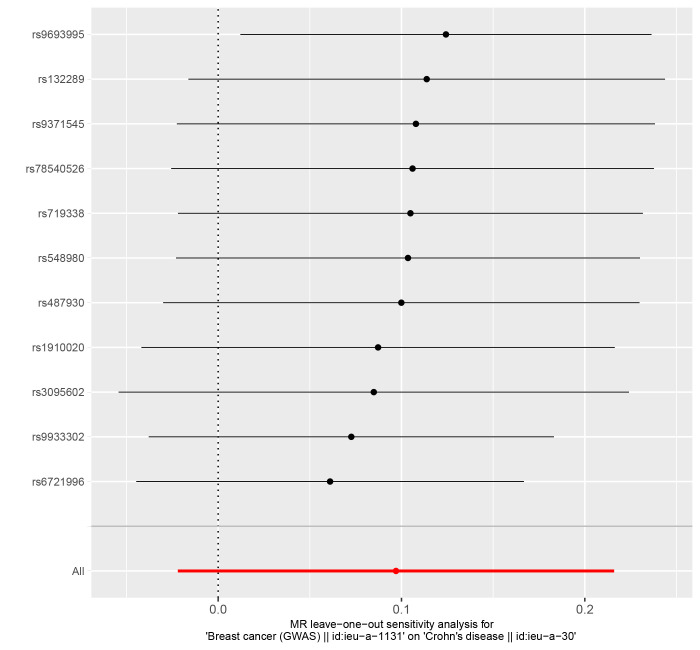


**Figure:**
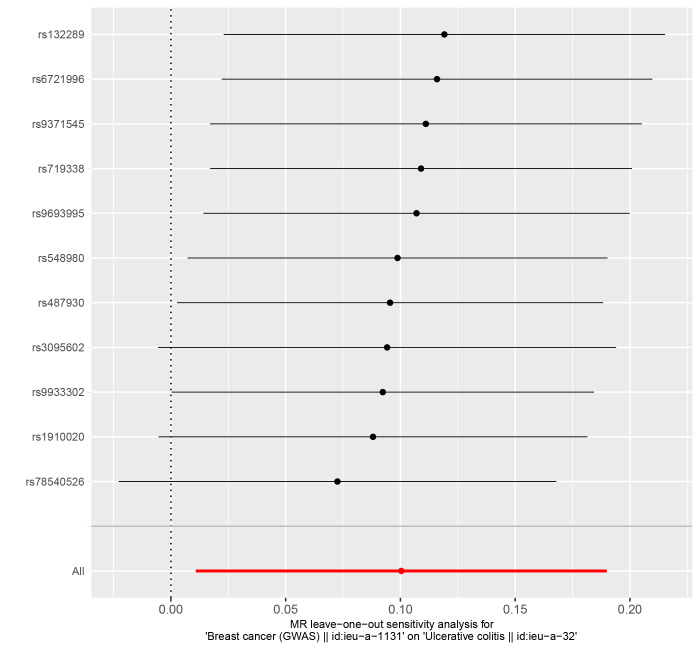


**Figure:**